# Mutations in genes encoding innate immune molecules identified in bladder cancer samples as potential biomarkers for immunotherapy with BCG and agonists

**DOI:** 10.3389/fruro.2023.984967

**Published:** 2023-03-20

**Authors:** Nina Marí Gual Pimenta de Queiroz, Fabio Mambelli, Bruno Marques Silva, Sergio Costa Oliveira

**Affiliations:** ^1^ Departamento de Bioquímica e Imunologia, Instituto de Ciências Biológicas, Universidade Federal de Minas Gerais, Belo Horizonte, Minas Gerais, Brazil; ^2^ Centro de Laboratórios Multiusuários (CELAM), Instituto de Ciências Biológicas, Universidade Federal de Minas Gerais, Belo Horizonte, Minas Gerais, Brazil; ^3^ Departamento de Imunologia, Instituto de Ciências Biomédicas, Universidade de São Paulo, São Paulo, Brazil

**Keywords:** bladder cancer, biomarkers, TCGA (The Cancer Genome Atlas Program), mutations, innate immunity

## Abstract

Bacillus Calmette–Guérin (BCG) immunotherapy for the treatment of bladder cancer (BC) depends on the recognition of bacteria by extracellular toll-like receptors (TLRs) or the detection of mycobacterial DNA by endosomal TLRs or the cGAS-STING pathway. Agonists related to these innate immune pathways have been developed as adjuvants to potentiate the effects of immunotherapy. As innate immune pathways are important for the action of BCG and other agonists proposed for BC therapy, we decided to investigate the presence of mutations in the main receptors of these pathways. The Cancer Genome Atlas (TCGA) database was screened to identify BC-related mutations (apart from oncogenes), targeting, in particular, TLRs, the adaptor molecule MyD88, and the cGAS-STING (cyclic GMP-AMP synthase-stimulator of interferon genes) immune pathway. Among 1,724 BC entries, 103 mutations were identified in 80 affected cases in the cohort. *TLR9* and *TLR10* ranked among the most frequent mutated genes observed in the affected cases in our search (13 mutations each). Through all analyzed data, the search for *MYD88* gene recovered only 1 mutation input in the database. Mutations in the *STING* and *cGAS* genes were found in one and four cases, respectively. We also evaluated clinical data, including the pathologic stage of BC, and gene expression from 103 mutations entries. This article attempts to highlight the relevance of mutations in genes coding for innate immune molecules in BC samples as potential biomarkers to predict individual disease outcome, and specifically to help find the appropriate treatment for each person in the future.

## Introduction

Urothelial bladder cancer (BC) is a heterogeneous malignancy with the potential to invade the different layers of the bladder wall. BC accounted for 3% of all new cases of cancer in 2020, and it was more frequently diagnosed in men (440,864 new cases) than in women (139,756 new cases) (1). The American Joint Committee on Cancer (AJCC) TNM system is often used to describe the stage of BC based on the results of physical examinations, biopsies, and imaging tests. BC can be described simply as invasive or non-muscle invasive bladder cancer (NMIBC). Pathologic stage T (i.e., T1 to T4b) describes how far the primary tumor has spread through the bladder wall and whether or not it has invaded adjacent tissues (2). Medical intervention depends on the pathologic stage of the disease. Invasive bladder tumors are typically treated by radical cystectomy. Patients with intermediate- or high-risk NMIBC are indicated for transurethral resection of the bladder tumor (TURBT) followed by intravesical instillations of bacillus Calmette–Guérin (BCG) and/or chemotherapy in order to prevent the risk of cancer progression or recurrence, according to the international guidelines (3–5). The numerous substrains of BCG are characterized by genetic and phenotypic differences, leading to distinct antitumor effects and variable clinical outcomes. The non-standardization of BCG manufacture and variation in the clinical protocols make the comparison among different substrains difficult. Several studies comparing overall survival and recurrence-free survival rates after treatment with different substrains in mice and humans are still not conclusive in defining the best BCG substrain. However, there is a concordance on the importance of maintenance therapy for a sustainable response and successful control of BC (6–9).

The use of BCG immunotherapy is still the most favorable intervention in terms of reducing NMIBC recurrence and progression. However, a significant number of patients (≈ 20%–30%) do not respond optimally to treatment, and others are resistant to BCG or subsequently relapse. BCG-unresponsive patients are also reported to have a poorer prognosis (10, 11). BCG action depends on both the immune response from normal urothelial cells and on the tumor cells, as they play an initial role in the recognition and processing of BCG for further activation of the immune response and subsequent modulation of the tumor microenvironment (TME), resulting in cytotoxicity against the cancer cells (12, 13). The recognition of BCG in the TME depends on the extracellular toll-like receptors (TLRs) or the detection of mycobacterial DNA by endosomal TLRs or the cGAS-STING (cyclic GMP-AMP synthase-stimulator of interferon genes) pathway (14–16). In order to improve treatment, agonists related to these innate immune pathways and other pathways have been developed as adjuvants (17–19). As it will be discussed, a wide range of innate immune pathways are important for BC treatment based on BCG and other agonists proposed as new therapies.

Despite efforts to define a panel of molecular biomarkers for risk stratification and treatment personalization, current BC management still relies on pathologic staging (20). Biomarkers could enable the use of a combination of methods, including histopathology, cystoscopy, urine cytology, and molecular urinary tests, to improve the sensitivity of BC diagnosis (21, 22). The Cancer Genome Atlas (TCGA) data have been explored as an important strategy to find the appropriate BC biomarkers (23–25). We aimed to analyze TCGA database, focusing on mutations in genes coding for innate immune molecules (i.e., MyD88, TLRs 1–10, cGAS, and STING) among 1,724 BC entries. We also evaluated clinical data including pathologic stages of BC and gene expression. The study involved only bioinformatic data analyses from human patients, which could suggest other investigations to understand mutations in innate immune molecules and help to determine prognosis and predict the outcome of disease treatment.

## Methods

The TCGA database was screened for BC entries. Searches targeted immunological genes of interest (i.e., those coding for the adaptor molecule MyD88 and TLRs 1–10, and the *STING*/*cGAS* genes) for mutations. The BC cases in which none of the selected genes presented mutations were collected as controls for the analyses. Data were collected using the data transfer tool API in the first week of April 2022 and were characterized in tables for further assessment. Two independent researchers collected and analyzed the data to avoid bias. Each case was characterized according to the following variables: case ID, mutation type (i.e., synonymous, non-synonymous), number of mutations, race (i.e., Asian, black, white, not reported), vital status (i.e., alive, dead), gender (i.e., male, female), AJCC pathological stage (i.e., 1–4b), and gene expression level. Synonymous and non-synonymous mutations (affected cases) were analyzed separately. Manifest files were generated from the filters, and files prefixed with “augmented_star_gene_counts.tsv” were used for gene expression analyses. Columns “tpm_unstranded” were used for extraction of expression values and “gene_name” were used for gene selection. Extracted data were treated using Python 3 libraries, Pandas, Seaborn, and GraphPad Prism. Refined data were plotted as grids, bars, heatmaps, and boxplot graphs for better visualization when fit.

## Results

### Mutations in innate immune molecules from human BC cases screened from the TCGA database

**Figure 1:**
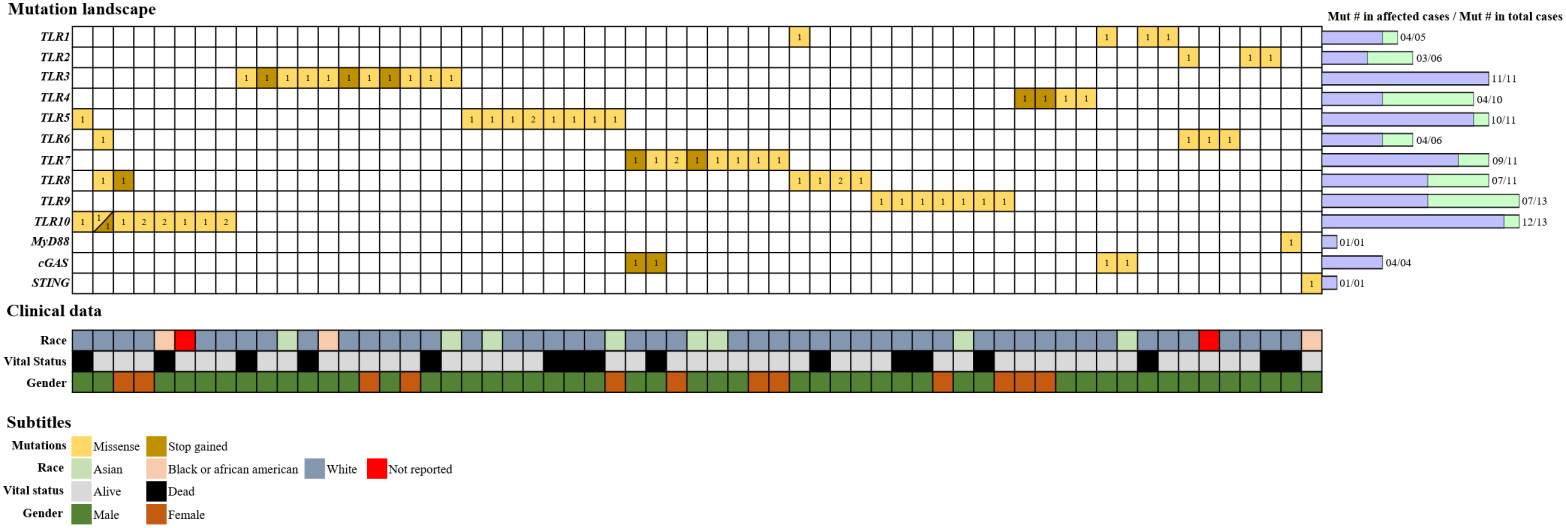
Alteration landscape of innate immune-related genes in BC. The TCGA database was screened for BC entries, and 103 somatic mutations were filtered in the innate immunological genes of interest from 80 cases. The affected cases accounted for 61 individuals, and each column on the grid represents a single case. Horizontal lines represent the genes, and the number of mutated copies is represented as registered inside the squares. Colors are used to differentiate missense mutations from stop–gained mutations. Somatic mutation numbers are represented by bars next to the grid. “Mut # in total cases” represents the total number of mutations identified per gene, whereas “Mut # in affected cases” represents the refined number of mutations per gene, including only non-synonymous mutations. Clinical data are reported above the grid for each case using a color scheme, and race, vital status, and gender are depicted.

The TCGA database was screened for BC entries in the first week of April 2022. As of the date of this study, TCGA contained publicly available datasets from 1,724 BC cases. The most frequently mutated genes in this cohort are *TTN* (titin) and *TP53* (tumor protein p53), which code for a protein abundant in striated muscles and for a tumor-suppressor protein, respectively. Despite the relevance of those highly mutated genes, our search was specifically targeted at mutations in genes that code for innate immune molecules, as these are important in the frontline of immune defense and are also related to some BC treatments. We screened 1,724 BC entries for mutations in the genes encoding TLRs 1–10 and MyD88, and in the *cGAS* and *STING* genes. BC cases in which none of the selected genes presented mutations were used as controls. Cases were characterized and screened for case ID, mutation type and number, race, vital status, gender, AJCC pathological state (1–4b), and gene expression level. From 103 mutations were identified in 80 patients in the analyzed cohort, 61 affected cases included non-synonymous mutations (missense and stop–gain mutations). The vast majority of the 61 affected cases (50/61, 82%) in the cohort were male. In terms of vital status, 74% of patients were alive and 26% were dead. The most frequently mutated genes in the cohort were *TLR9* and *TLR10*, with 13 somatic mutations each, followed by *TLR3*, *TLR5*, *TLR7*, and *TLR8*, with 11 mutations each, and *TLR4*, with 10 mutations. A search for mutations in other important innate immune molecules uncovered only one mutation related to the *MyD88* gene and one and four related to the *STING* and *cGAS* genes, respectively (**Figure 1**).

**Figure 2:**
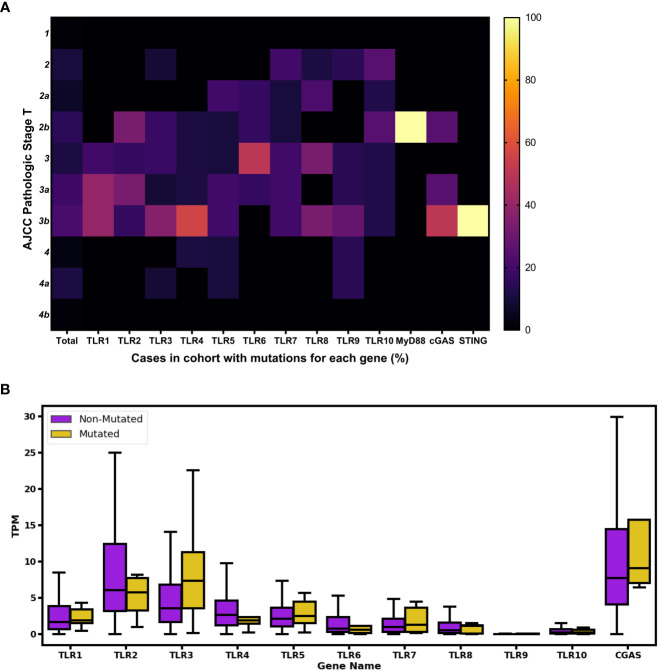
Phenotypical impact of the analyzed innate immune-mutated genes in human BC cases. **(A)** The pathological stage of the total affected cases was assessed by screening AJCC clinical pathologic stage T data. Color scale ranges from 0% (shown in black) to 100% (shown in bright yellow), indicating the percentage of normalized number of mutations according to 1–4b pathological stages. For comparison purposes, the “Total” column represents the totality of BC entries in TCGA without mutations in the analyzed genes, and the percentage number for each pathological stage is depicted. **(B)** Boxplot showing gene expression distribution using TPM values. In purple are represented cases in which none of the selected genes were mutated; in yellow are represented cases with *TLR*s 1–10 and *cGAS* genes with mutations. Outliers were removed for better fit and visualization.

We also evaluated the clinical data, including pathologic stages of BC and gene expression from the 103 mutations. In order to analyze the distribution of AJCC pathologic stage T disease among the cases, we compared mutated cases in the analyzed genes and the totality of BC entries in TCGA without mutations. After analyzing the percentage of cases of each stage using heatmap, we observed that a few of the total cases (without mutations) were described as being at stage 1 or 4, and most were equally distributed between stages 2 and 3. However, when we evaluated the cases with mutations in different *TLR* (1, 2, 3, 4, 6, 8, and 9) genes, we noticed a tendency toward an increase in the percentage of cases in stage 3 (i.e., stages 3, 3a, and 3b). The *MyD88* and *STING* genes were each mutated in only one case, described as stage 2b and 3b, respectively. Finally, among the four cases with mutations in the *cGAS* gene, two were described as stage 3b, one as stage 3a, and other as stage 2b (**Figure 2A**). Comparison of gene expression between total non-mutated cases and those mutated in the genes of interest shows a tendency for a decrease in the expression of the *TLR2*, *TLR4*, and *TLR6* mutated genes and an increase in the expression of the *TLR3* mutated gene (**Figure 2B**).

### Mutations in innate immune molecules with potential as biomarkers for BC

BCG immunotherapy has been recommended in NMIBC cases for almost 50 years and been preconized at least six successive instillations of BCG after TURBT. A study analyzed the immune response following six intravesical administrations of BCG. The study showed that the highest detection of TNF alpha (TNF-α) in peripheral blood monocytes of patients was after the fourth administration of BCG (26). Activation of the immune response in the BCG bladder is progressive, suggesting that there may be an induction of immune memory (27). In this study, all the mutated cases were related to the invasive stages, and the altered expression of these innate immune genes highlights the feasibility of studying them as biomarkers for disease progression. It also tests our current understanding as to how much of an impact they would afford regarding the impairment of BCG immunotherapy in muscle-invasive BC. Our main goal was to investigate possible mutations in molecules that could interfere with the response to BCG, which could also be related to other agonists proposed for cancer immunotherapy. The fact that we did not find mutations in the samples from patients with stage T1 disease, for which treatment with BCG is recommended, may be a good indicator for the success of BCG in these cases. However, the numbers of cases with the disease in the initial stage, that is, stage T1, or with more severe disease, that is, stage T4, were very small; in contrast, the majority of cases were from patients with intermediate-stage disease, stage T2 and stage T3. Therefore, the differences in the number of samples did not allow us to reach final conclusions.

Different innate immune pathways in both normal epithelial and cancer cells are essential for BCG recognition, processing, and the subsequent induction of the antitumor response (12, 13). Mycobacterial lipoproteins and DNA are recognized by TLR2 and TLR9, respectively (28–31); TLR4 is required to activate a robust adaptive response (28, 32). TLRs act as an important interface between innate and adaptive immunity. BCG-specific responses influence the cell infiltrate profile in the TME by specifically recruiting effector cells such as CD8+ cytotoxic T cells (CTLs) and inflammatory macrophages (13, 33–35). All TLRs signal through the MyD88 pathway, except for TLR3, which recruits TRIF, and TLR4, depending on both MyD88 and TRIF. The MyD88 pathway activates MAPKs and NF-κB, whereas TRIF induces IRF3 and, consequently, type I IFN production (15). The cGAS-STING pathway comprises other important innate immune pathways induced by cytosolic DNA that also lead to type I IFN release (16, 36).

Our group has previously investigated the relevance of several innate immune molecules in a subcutaneous mouse bladder tumor model (MB49) treated with BCG. We used different knockout mice (TLR2^–/–^, TLR3^–/–^, TLR4^–/–^, TLR7^–/–^, TLR9^–/–^, MyD88^–/–^, STING^–/–^, cGAS^–/–^, IFNAR^–/–^, IL-1R^–/–^, caspase 1/11^–/–^, and gasdermin D^–/–^); we found that only MyD88 was essential for tumor regression and important for the induction of cellular infiltrate and inflammatory profile modulation in the TME in response to BCG intratumoral treatment. The intrinsic response of MB49 cancer cells to BCG was not relevant, but we hypothesized that BCG immunotherapy activates the extrinsic innate immune response in immune cells present in the TME, inducing inflammatory macrophages and leading to a subsequent specific adaptive response (35). Therefore, in addition to considering the importance of mutations in cancer cells, it is also imperative to evaluate their effects on the function of other immune cells present in the TME. It is also important to consider that, although some genes such as *MyD88*, *STING*, and *cGAS* presented fewer mutations than TLRs in this screening, we did not evaluate possible epigenetic alterations. *cGAS* and *STING* were analyzed in human cell lines and tissue samples from different types of cancer without important genome mutations, but, interestingly, the cGAS-STING pathway is often suppressed in a variety of cancers by epigenetic mechanisms involving DNA hypermethylation (37–39).

### Biomarkers for BC treatments using BCG or other innate immune agonists

In addition to the importance of these innate immune molecules as potential biomarkers of disease severity, we also need to consider these mutations as prognostic biomarkers for BC treatments using BCG or other innate immune agonists. Therapeutic intratumoral administration of STING adjuvants controls different tumor models, presumably through direct activation of STING in the TME, leading to activation of the immune response driven by dendritic cell-dependent CTLs (40–42). There have been many attempts to activate an efficient antitumor response using STING agonists (43, 44). Another strategy proposed a recombinant BCG-overexpressing STING agonist (c-di-AMP), inducing a robust inflammatory macrophage profile and trained immunity response in the BC murine model (19). TLR agonists have been developed as candidates to improve BC therapy, like Poly(I:C) that activates type I IFN *via* TLR3 (45, 46), Polyporus polysaccharide (PPS) that activates macrophages through the TLR4/NF-κB signaling pathway (47), TMX-101 and TMX-102 that acts *via* TLR7 (17, 48), and CpG that induces TLR9 (18) and others (14, 49). TLR10 is the least understood member of the TLR family that forms dimers with other TLRs. TLR10 ligands are still being studied, and it could be useful to modulate both pro- and anti-inflammatory responses (50, 51). TLR agonists should also be considered as potential activators of BCG-induced innate immune memory (52).

The majority of the studies evaluate TLR activation markers indirectly by measuring cytokines in urine or blood, and very few studies have evaluated the expression of TLRs in cancer cells after stimulation with BCG or other agonists (53) or in patient samples. The expression levels of TLR4, TGF-β1, IFN-γ, and TNF-α were evaluated in samples of urinary bladder tumors and unaffected adjacent tissue from 50 patients. The results showed that cancer cells are characterized by lower expression levels of TLR4, TGF-β1, IFN-γ, and TNF-α, and that TLR4 expression was identical in low- and high-grade cancer (54). However, another robust study that analyzed the expression of TLRs 1–10 in 24 urothelial cancer samples and 46 non-tumoral bladder tissue samples found the opposite, with increased expression of TLRs 2–7 and TLR10 in the cancer samples. It also found that IL-1β, IL-6, and IL-8 cytokine levels were higher in urine from cancer patients, but that TLR expression levels in the urine of cancer patients were the complete opposite of those seen in tissue samples (55). The use of TLRs as biomarkers still poses a challenge and requires further investigation in different pathologic stages and treatments.

Evaluation of innate immune pathways in cancer might be considered for the development of novel cancer immunotherapies. We evaluated 1,724 BC entries from TCGA, identifying 103 mutations in 80 affected cases in the cohort. *TLR9* and *TLR10* ranked among the most frequently mutated genes. Comparison of gene expression profiles between all non-mutated cases and those mutated in the genes of interest shows a tendency for a decrease in the expression of the *TLR2*, *TLR4*, and *TLR6* mutated genes and an increase in the expression of the *TLR3* mutated gene. Although the *TLR9* and *TLR10* genes presented the largest number of mutations, the gene expression from these receptors were not significantly different. The limitations of our study include the fact that it involved only bioinformatic analyses of data from human patients available in the TCGA database. Another limitation was that we could not correlate TCGA mutations and BCG treatment, since we had no access to all the data from these patients treated with BCG and we could not assume that they were treated with the same standardized protocols. We also reinforce the necessity to correlate gene mutations, protein expression, and epigenetic modifications for future studies. However, our results highlight the importance of future experimental studies to confirm these mutations as biomarkers in predicting individual disease outcomes and for clinical implications with BCG and other agonists proposed as adjuvants for BC therapy.

## Data availability statement

Publicly available datasets were analyzed in this study. The TCGA bladder cancer cohort data from the TCGA database is available at https://www.cancer.gov/tcga.

## Author contributions

NQ, FM, and SO conceptualized this study. NQ and FM were in charge of data curation and formal analysis. BS performed the bioinformatics analyses. NQ, FM, and SO wrote the original draft and the final manuscript. All authors contributed to the article and approved the submitted version.
